# Arterial Oxygen Content Is Precisely Maintained by Graded Erythrocytotic Responses in Settings of High/Normal Serum Iron Levels, and Predicts Exercise Capacity: An Observational Study of Hypoxaemic Patients with Pulmonary Arteriovenous Malformations

**DOI:** 10.1371/journal.pone.0090777

**Published:** 2014-03-17

**Authors:** Vatshalan Santhirapala, Louisa C. Williams, Hannah C. Tighe, James E. Jackson, Claire L. Shovlin

**Affiliations:** 1 Imperial College School of Medicine, Imperial College, London, United Kingdom; 2 National Heart and Lung Institute (NHLI) Cardiovascular Science, Imperial College, London, United Kingdom; 3 Respiratory Medicine, Hammersmith Hospital, Imperial College Healthcare NHS Trust, London, United Kingdom; 4 Department of Imaging, Hammersmith Hospital, Imperial College Healthcare NHS Trust, London, United Kingdom; National Institute of Child Health and Human Development, United States of America

## Abstract

**Background:**

Oxygen, haemoglobin and cardiac output are integrated components of oxygen transport: each gram of haemoglobin transports 1.34 mls of oxygen in the blood. Low arterial partial pressure of oxygen (PaO_2_), and haemoglobin saturation (SaO_2_), are the indices used in clinical assessments, and usually result from low inspired oxygen concentrations, or alveolar/airways disease. Our objective was to examine low blood oxygen/haemoglobin relationships in chronically compensated states without concurrent hypoxic pulmonary vasoreactivity.

**Methodology:**

165 consecutive unselected patients with pulmonary arteriovenous malformations were studied, in 98 cases, pre/post embolisation treatment. 159 (96%) had hereditary haemorrhagic telangiectasia. Arterial oxygen content was calculated by *SaO_2_ x haemoglobin x 1.34/100*.

**Principal Findings:**

There was wide variation in SaO_2_ on air (78.5–99, median 95)% but due to secondary erythrocytosis and resultant polycythaemia, SaO_2_ explained only 0.1% of the variance in arterial oxygen content per unit blood volume. Secondary erythrocytosis was achievable with low iron stores, but only if serum iron was high-normal: Low serum iron levels were associated with reduced haemoglobin per erythrocyte, and overall arterial oxygen content was lower in iron deficient patients (median 16.0 [IQR 14.9, 17.4]mls/dL compared to 18.8 [IQR 17.4, 20.1]mls/dL, p<0.0001). Exercise tolerance appeared unrelated to SaO_2_ but was significantly worse in patients with lower oxygen content (p<0.0001). A pre-defined athletic group had higher Hb:SaO_2_ and serum iron:ferritin ratios than non-athletes with normal exercise capacity. PAVM embolisation increased SaO_2_, but arterial oxygen content was precisely restored by a subsequent fall in haemoglobin: 86 (87.8%) patients reported no change in exercise tolerance at post-embolisation follow-up.

**Significance:**

Haemoglobin and oxygen measurements in isolation do not indicate the more physiologically relevant oxygen content per unit blood volume. This can be maintained for SaO_2_ ≥78.5%, and resets to the same arterial oxygen content after correction of hypoxaemia. Serum iron concentrations, not ferritin, seem to predict more successful polycythaemic responses.

## Introduction

The primary function of haemoglobin is to transport oxygen from the alveolar capillaries to the tissues. Blood oxygen content is determined by the haemoglobin concentration, and the partial pressure of oxygen in blood (PaO_2_) which governs the percentage haemoglobin saturation (SaO_2_) [1]. In turn, the overall transport of oxygen to the tissues depends upon the oxygen content of arterial blood, and the volume of blood reaching the tissues in any given period (cardiac output) [1]. Profound arterial hypoxaemia can be tolerated if subjects are gradually acclimatised [2] [3] [4] [5]. Mechanisms that help sustain oxygen delivery include higher haemoglobin concentrations through secondary erythrocytosis/polycythaemia [2] [3] [4], and tissue changes that optimise energy metabolism including suppression of mitochondrial biogenesis and oxidative metabolism[5]. Similarly, severe anaemia is tolerated if chronic compensatory mechanisms can be employed: anaemic patients unable to sustain normal haemoglobin concentrations have hyperdynamic circulations, with high cardiac outputs accompanied by lower systemic vascular resistance [6] [7] [8].

Although this is an integrated system of oxygen delivery, and challenged by multiple different pathological states, the problem is that components are generally discussed in a discipline-restricted manner. Oxygen and clinical practice guidelines are based on PaO_2_ and/or SaO_2_ measurements: of more than 2,700 PubMed results retrieved using “oxygen” and “guidelines,” only 106 were found by including the term “haemoglobin” [or ”hemoglobin”], with relevant articles predominantly restricted to the anaesthetic/critical care literature. Synthesis of iron-containing haemoglobin is impaired by iron deficiency which affects more than a billion individuals worldwide[9], but in the iron deficiency literature, anaemia is generally discussed without reference to oxygen transport: On 25.11.2013 although there were 14,284 PubMed references for “iron”, “deficiency” and “anaemia/anemia,” only 73 were retrieved including the terms “oxygen” and either “delivery” or “transport.” In addition, it is rarely discussed that hypoxaemia caused by low inspired oxygen concentrations, at altitude [2] [3] [4] [5], or due to alveolar/airways disease [10], is inevitably accompanied by a separate process, namely hypoxic pulmonary vasoconstriction (HPV) [2] [11]. HPV is the physiological response to alveolar hypoxia, responsible for ventilation-perfusion matching [2] [11]. As HPV leads to local or generalised elevation of pulmonary vascular resistance [2] [11], this process has the potential to confound physiological and clinical responses in hypoxaemic subjects.

Hypoxaemic patients with pulmonary arteriovenous malformations (PAVMs) [12] provide an opportunity to study integrated oxygen/haemoglobin relationships without confounding hypoxic pulmonary vasoreactivity: PAVMs are abnormal blood vessels that usually develop by teenage years, and provide direct capillary-free communications between the pulmonary and systemic circulations [12]. Hypoxaemia results from deoxygenated pulmonary arterial blood transiting these right-to-left (R-L) shunts and bypassing the alveolar capillary sites of gas exchange. There is good agreement between the calculated R–L shunt using measured SaO_2_, and the anatomic R-L shunt, confirming that the R-L shunt is the predominant cause of the arterial hypoxaemia [13]. Hypoxaemic PAVM patients are therefore not at risk of hypoxic pulmonary hypertension, and pulmonary vascular resistance at rest is low in patients with severe PAVMs [14] [15]. Arterial PaO_2_ and SaO_2_ are inversely related to the proportion of the cardiac output passing through the R–L shunts [13] [14] [15] [16] [17] [18] [19] [20] [21] [22] [23] [24], and hypoxaemia may be severe. As at altitude, and in patients with other cardiorespiratory cyanotic disease, secondary polycythaemia [13] [14] [15] [16] [17] [18] [19] [20] [21] [22] [23] [24] [25] and increased cardiac outputs [14] [19] help to sustain long term oxygen delivery in hypoxaemic PAVM patients.

PAVM patients provide a particularly good model in which to study integrated oxygen/haemoglobin relationships not only because of the absence of HPV, but also because serial evaluations can be performed before and after correction of hypoxaemia by PAVM embolisation treatments which are recommended to prevent paradoxical embolic strokes and other complications [23] [24] [25] [26] [27]. High proportions of PAVM patients have iron deficiency, due to the presence of underlying hereditary haemorrhagic telangiectasia (HHT), [28] [29], and inadequate replacement of haemorrhagic iron losses [30] [31] [32]. Importantly, such iron deficiency generally occurs in the absence of confounding inflammation: inflammatory markers such as C reactive protein are generally normal [24] [33], and hepcidin concentrations are appropriate for iron stores, exhibiting the same relationships with ferritin as in the general population [30].

Our aim was to evaluate oxygen/haemoglobin relationships in a large cohort of PAVM patients before and after embolisation treatment, and in the presence and absence of iron-restricted erythropoesis, in order to obtain insights not available from other hypoxaemic pathologies or models. This is important for PAVM patients because they are currently managed by oxygen/polycythaemia guidelines extrapolated from evidence in other patient groups [34] [35] [36], and our clinical observations suggested that the extrapolated guidelines were not serving the population well [12] [37].

The study objectives were achieved. Here we present data that provide new insights into the regulation of blood oxygen content through polycythaemia, and add to the evidence base from which clinical guidance may be developed.

## Methods

Ethical approved was from the Hammersmith, Queen Charlotte's, Chelsea, and Acton Hospital Research Ethics Committee (LREC 2000/5764: “ Case Notes Review: Hammersmith Hospital patients with pulmonary arteriovenous malformations and hereditary haemorrhagic telangiectasia (HHT).”) The ethics committee approved the review of the case notes for research purposes without seeking individual consents. Individuals were asked for written consent as part of separate ethics approvals for any research protocol (questionnaire, blood test, scan or other study) that was not part of standard clinical practice, but no such data are reported in this manuscript.

### Clinical evaluations

Due to the pan-UK referral base to our PAVM/HHT service, patients are initially evaluated in single day assessments (see www.imperial.ac.uk/nhli/hht_pavm_patient). For patients referred with suspected/confirmed HHT [38] [39], PAVM screening or assessment is performed using validated SaO_2_ measurements [24], and thoracic CT scans if not performed previously/recently. The study cohort represents the 165 consecutive patients with CT-proven PAVMs first seen between June 2005- September 2010, and excludes the May 1999-May 2005 patients whose findings [23] precipitated the current study.

#### Presentation assessments

At initial assessment, SaO_2_ was measured by pulse oximetry (Ohmeda Biox 3900, Boulder, Colorado) while breathing room air. Measurements were made for 10 minutes in the erect posture, recorded at one minute intervals, with the mean value from minutes 7–10 reported, as utilised in previous clinical studies [17] [20] [21] [23] [24] [33] [40] and recently validated [24]. To minimise diurnal variability for iron measurements, timings of blood tests were standardised to mid-late afternoon [33]. Full blood counts measured haemoglobin, haematocrit, red blood cell (RBC, erythrocyte) number, mean corpuscular haemoglobin (MCH), mean corpuscular haemoglobin concentration (MCHC) and red cell distribution width (RDW)) on XE Series Analysers (Sysmex, UK). Biochemical indices including serum iron, transferrin saturation index (T*f*SI) and ferritin were measured on Ci1600 Architect Analysers (Abbott Diagnostics, Ireland).

#### Embolisation admissions and follow up

Embolisation was performed as described elsewhere [12] [37] [40] [41] in 98 patients during standardised 48hr admissions. Pulmonary artery pressures (PAP) were recorded by a centrally-placed catheter prior to contrast injection [12] [37] [40] [41]. Embolisation was not indicated in 66 patients, primarily due to PAVMs with feeding arteries too small for treatment (n = 63 including two who had previous maximal treatment elsewhere). Three patients had contraindications [40] [42], including two with very severe pulmonary hypertension, one of whom was on a liver transplant waiting list. One patient declined embolisation. Twenty patients required more than one planned embolisation session to achieve maximal embolisation; seventeen required two sessions which spanned 1.5–13 (median 4.3) months; three required three sessions spanning 19–24 (median 20) months. Oximetry and blood tests were repeated in the 24hs before each pulmonary angiography/embolisation session, and oximetry the day after embolisation. Oximetry and blood tests were repeated in a standardised follow-up clinic which took place at least 2 months after the final embolisation. In their first post-embolisation follow up clinic, treated patients were asked in a non biased manner whether they had noticed any differences after embolisation, then asked to report what those differences were.

### Study methodology

Pre-study power calculations to determine series length were performed on the HHT thrombotic endpoints under parallel study [23] [24] [33] [43]. Blinded to all other measurements (and subsequent analyses), two investigators (VS, CLS) assigned patient's clinic reports of their presentation exercise capacity to a scale [44] adapted from the Medical Research Council dyspnoea scale [45]. Because many were highly athletic individuals, the “normal” Grade 1 classification was subclassified into Grade 1a (if participating in intense sporting activity such as rowing, football, distance cycling or gym activities at least three times per week), and Grade 1b (representing other normals, who described dyspnoea only on strenuous exertion). Grades 2–5 describe progressively lower exercise tolerance/greater dyspnoea. The grading of individuals in the current cohort was published in Abstract format in 2011 [44]. Treated patients were also stratified as “improved exercise tolerance” or “no improvement” by their reports of changes post embolisation, blinded to other patient variables. Study follow up continued until all feasible first post embolisation assessments had been performed. In order to avoid inadvertent introduction of bias, other than entering data for remaining post embolisation assessments, no additional information was sought, no additional assessments were performed for the purposes of this study, and specifically, groupings generated by exercise tolerance or subsequent statistical analyses were not restudied or reassessed.

For the current study, oxygen content of blood and iron deficiency were generated as new variables by command line entry, blinded to all other parameters. Arterial oxygen content was calculated by *SaO_2_ x haemoglobin x 1.34/100* where SaO_2_ was expressed as a %, and 1.34mls is the amount of oxygen carried per gram of haemoglobin [1]. Iron deficiency was assigned as absent (“0”) if ferritin, iron and T*f*SI were all clearly in the normal range (ferritin>20 µg/L, serum iron>11 µmol/L and T*f*SI>20%). In keeping with current recommendations [46] [47] [48], iron deficiency was assigned as present (“1”) if same-day ferritin was <15 µg/L. As discussed previously by ourselves [24] [33] and others [46] [47] [48], ferritin may be spuriously high in iron deficiency due to concurrent pathologies. Thus following haematinic validations [24], iron deficiency was also assigned as present (“1”) for individuals with both iron and T*f*SI clearly subnormal (<7 µmol/L and <20% respectively). All other combinations were assigned as intermediate/unknown (“.”).

### Statistical Analyses

STATA IC version 12 (Statacorp, Texas) and GraphPad Prism 5 (Graph Pad Software Inc, San Diego) were used to calculate distributions of participant-specific variables, to perform comparisons between groups, and to generate graphs. Two group comparisons were by Spearman rank or Mann Whitney; three group repeated measures comparisons by Kruskal Wallis with post-test Dunns corrections. Univariate and multivariate linear, logistic and quantile regression was performed in STATA IC version 12 (Statacorp, Texas).

## Results

### Series demographics

The 165 patients were aged 17–87 (median 49) years. Sixty-two (37.6%) were male. 159 (96.4%) patients had underlying hereditary haemorrhagic telangiectasia (HHT) [38]. PAVMs had been diagnosed by a variety of routes, most commonly screening for PAVMs in suspected HHT patients/families (N = 62 [37%]); incidental detection by chest x-rays or thoracic/abdominal CT scans (N = 34 [20.5%]); investigations following strokes, brain abscess or neurological symptoms (N = 18 [12%]); and PAVM respiratory symptoms (N = 17 [10%]). Only 15 (9.1%) patients had evidence of significant co-existing disease, with obstructive spirometry due to either asthma or COPD the most common. One patient was receiving supplementary oxygen therapy at presentation and in follow up. No patients underwent venesection in the course of these studies.

SaO_2_ at rest ranged from 78.5–99% (median 95%, Table 1). In keeping with the high prevalence of HHT, many of the PAVM population had biochemical and haematinic evidence of iron deficiency (Table 1). Overall, haemoglobin ranged from 7.7 to 20.9 g/dl (median 14.1 g/dl), haematocrit from 0.26 to 0.61 (median 0.43).

**Table 1:** Demographics of 165 consecutive unselected PAVM patients.

*Continuous variables*	*N*	*Range*	*Median*	*IQR*
Age (yr)	165	17–87	49	36·5, 62·5
SaO_2_ at presentation (%)†	165	78·5–99	95	92, 96
Pulse at presentation (min^−1^) †	164	58·5–123	88	78·8, 98·5
Haemoglobin (g/dl)	160	7·7–20·9	14·1	12·8, 15·6
Haematocrit (%)	159	0·26–0·61	0·43	0·40, 0·46
Red blood cell (RBC) count	159	2·87–7·65	4·9	4·6, 5·2
MCH, pg/cell	159	16·5–35·4	29·8	28, 31·3
MCHC, g/dl	159	26·6–36·7	33·5	32·2, 34·3
MCV, fl	160	62·1–104	88·4	85·5,92·6
Serum iron (µmol/l)Δ	141	1–55	14	9, 19·5
Transferrin saturation index (T*f*SI%)Δ	138	0–71	24	15, 32
Ferritin (µg/L)	105	3–409	34	19·5, 68·5

**Demographics of the 165 PAVM Patients**. N, number of datasets: values <165 imply that data was not available for a subgroup of patients. †SaO_2_ measured continuously at rest for 10 minutes standing breathing room air, with recordings at one minute intervals, and reported values the mean of the readings after 7, 8, 9 and 10 minutes. MCH, mean corpuscular haemoglobin. MCHC, mean corpuscular haemoglobin concentration. MCV, mean corpuscular volume. Δ Blood test timings were standardised to catch the afternoon peak in iron levels as described in the online supplement to Livesey et al. [33].

### Basis of polycythaemia and anaemia responses

**Figure 1:**
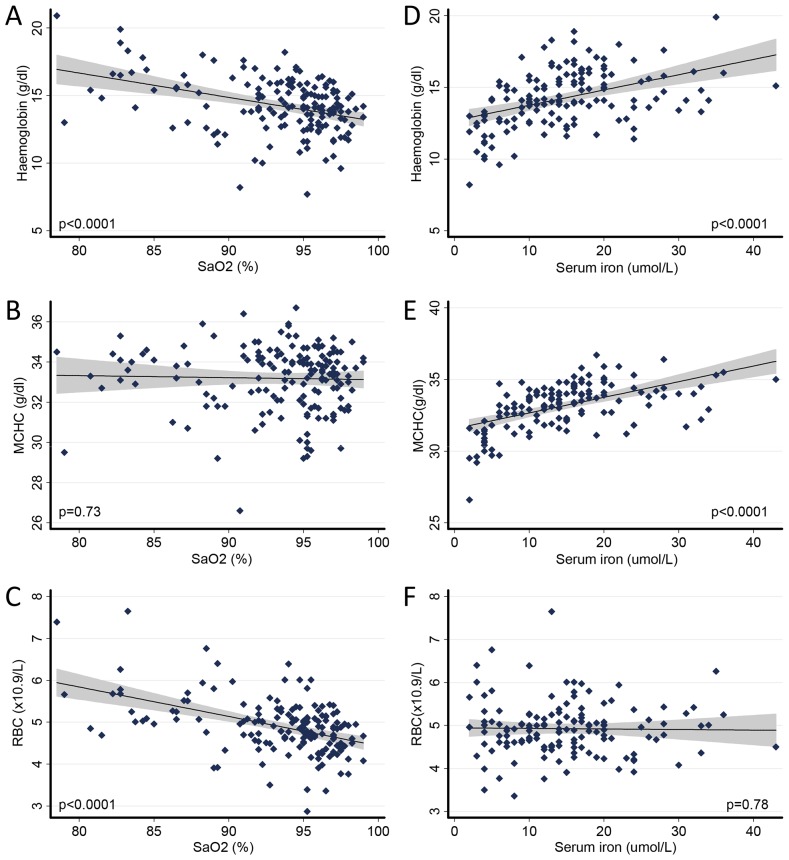
Basis of polycythaemia and anaemia responses in PAVM patients. Univariate associations demonstrating individual patient data (small diamonds), linear regression lines, and 95% confidence intervals (shaded) for two way relationships. A–C): The polycythaemic response to hypoxaemia. A) Lower SaO_2_ was associated with higher haemoglobin (the ‘polycythaemic response’). B) This polycythaemia was not attributable to increased haemoglobin concentration in red cells (mean corpuscular haemoglobin concentration, MCHC). C) Instead, the polycythaemia reflected increased red cell number (RBC), that is, secondary erythrocytosis, D–F) The anaemic response to iron deficiency: D) Lower serum iron concentrations were associated with lower haemoglobin (the ‘anaemic response’). E) This anaemic response to iron deficiency resulted from reduced haemoglobin concentration in red cells (MCHC), and not a change in red cell number (RBC) (F).

As expected, haemoglobin values were higher in patients with lower SaO_2_ (Figure 1A). On average, for every 1% fall in SaO_2_, haemoglobin rose by 0.82 g/dl (regression coefficient -0.82 (95% CI −1.12, −0.51, p<0.0001). There was no change in the haemoglobin concentration per red cell (Figure 1B), and the rise in haemoglobin reflected higher red cell counts at lower SaO_2_ (Figure 1C). In the same population, lower serum iron was associated with lower haemoglobin (Figure 1D), attributable to a reduced haemoglobin concentration per red cell, with no change in red cell count (Figure 1E, 1F).

**Figure 2:**
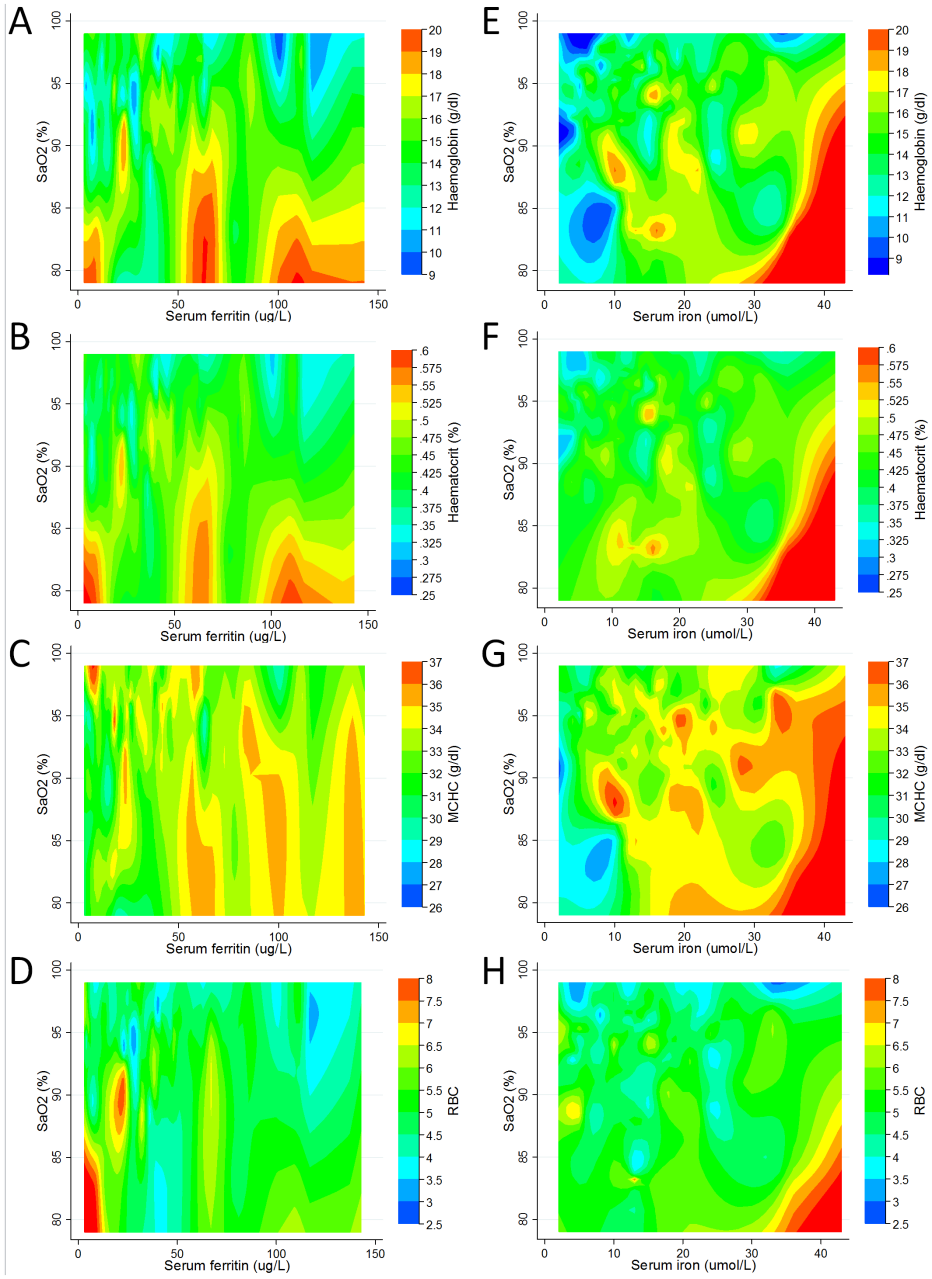
Three-way plots of relationships between haematinic indices and SaO_2_. Three-way plots of relationships between SaO_2_ (y axis), ferritin/iron (x axis), and haematinic index (z axis). The most hypoxaemic patients are at the bottom of each graph, and patients with the lowest ferritin/iron levels to the left. The z axis effectively provides a 3 dimensional plot in which contours range from blue (lowest value of modelled haematinic variable) to red (highest values). A–D) Serum ferritin/SaO_2_ stratifications for the 105 PAVM patients with serum ferritin measurements: A) SaO_2_/ferritin/haemoglobin. B) SaO_2_/ferritin/haematocrit. C) SaO_2_/ferritin/MCHC. D) SaO_2_/ferritin/red cell count. Note that higher haemoglobin, haematocrit and MCHC are seen across the normal range for ferritin (10–150 or 20–300 µg/L, according to gender), but higher RBC number is only seen in patients with low or subnormal ferritin. E–H) Serum iron/SaO_2_ stratifications for the 141 PAVM patients with serum iron measurements. E) SaO_2_/iron/haemoglobin. F) SaO_2_/iron/haematocrit. G) SaO_2_/iron/MCHC. D) SaO_2_/iron/red cell count. Although serum iron and serum ferritin were correlated (Spearman rho 0.34, p = 0.006), serum iron did not show the same relationships with hematinic indices as serum ferritin (contrast A and E; B and F; C and G; D and H). The highest haematinic indices were observed with serum iron above the normal range (7–27 µmol/L).

To portray the inter-relationships graphically, three-way contour plots were generated. These indicated that the polycythaemic response was evident even in the setting of low ferritin concentrations: For haemoglobin (Figure 2A), haematocrit (Figure 2B), and MCHC (Figure 2C), higher values were observed in more hypoxaemic patients across all ferritin values. The erythrocytotic response in hypoxaemic patients was particularly prominent in patients with subnormal serum ferritin (Figure 2D).

Different haematinic/SaO_2_ inter-relationships were observed with serum iron. Three-way contour plots indicated that for patients with lower SaO_2_, polycythaemic responses (higher haemoglobin (Figure 2E), higher haematocrit (Figure 2F), higher MCHC (Figure 2G) and higher red cell count (Figure 2H)) were evident only in the setting of high-normal serum iron concentrations.

### Preservation of arterial oxygen content by polycythaemic response

**Figure 3:**
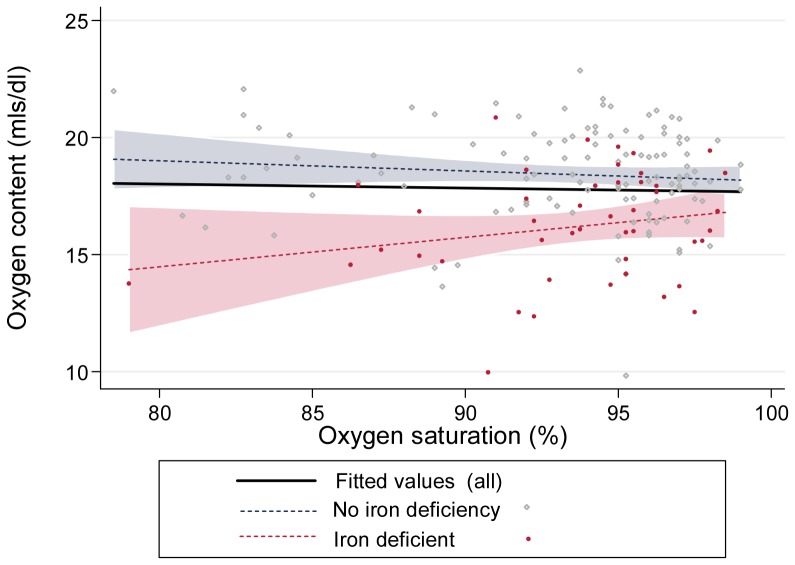
Arterial oxygen content stratified by oxygen saturation (SaO_2_). Oxygen content across all degrees of hypoxaemia, calculated by *SaO_2_ x haemoglobin x 1.34/100*, where SaO_2_ was expressed as a %, and 1.34mls is the amount of oxygen carried per gram of haemoglobin.[1] The bold black line represents the regression line for all patients, irrespective of iron status (p = 0.69). Grey diamonds/dotted line/shaded 95% confidence interval represent patients without iron deficiency (p = 0.33). Red diamonds/dotted line/shaded 95% confidence interval represent patients with iron deficiency (p = 0.97).

Due to the higher haemoglobin in more hypoxaemic patients, oxygen content per unit blood volume was similar across all degrees of hypoxaemia (bold black line, Figure 3). SaO_2_ explained only 0.1% of the variance in arterial oxygen content per unit blood volume (p = 0.83). In non iron deficient patients, the median arterial oxygen content was 18.8 [IQR 17.4, 20.1]ml/dl. Iron deficient patients had similar SaO_2_ to non iron deficient patients, but haemoglobin was ∼2 g/dl lower. As a result, arterial oxygen content in iron deficient patients either defined conventionally by ferritin <15 µg/L (median 16.0 [IQR 14.9, 17.4]ml/dl) or by “low iron state” which included patients with serum iron <11 µmol/L irrespective of ferritin (median 16.3 [IQR 14.8, 18.1]ml/dl), were both substantially lower than in non iron deficient patients (p values<0.0001, Figure 3).

### Consequences of reduced oxygen content

The median values for arterial oxygen content (18.8 ml/dl versus 16.0 ml/dl, see Figure 3) imply that per unit blood volume, oxygen content was reduced by approximately 15% in the iron deficient group. Graded self-reported exercise tolerance [44] was used as a crude indicator of whether compensating for reduced blood oxygen content was achievable.

**Figure 4:**
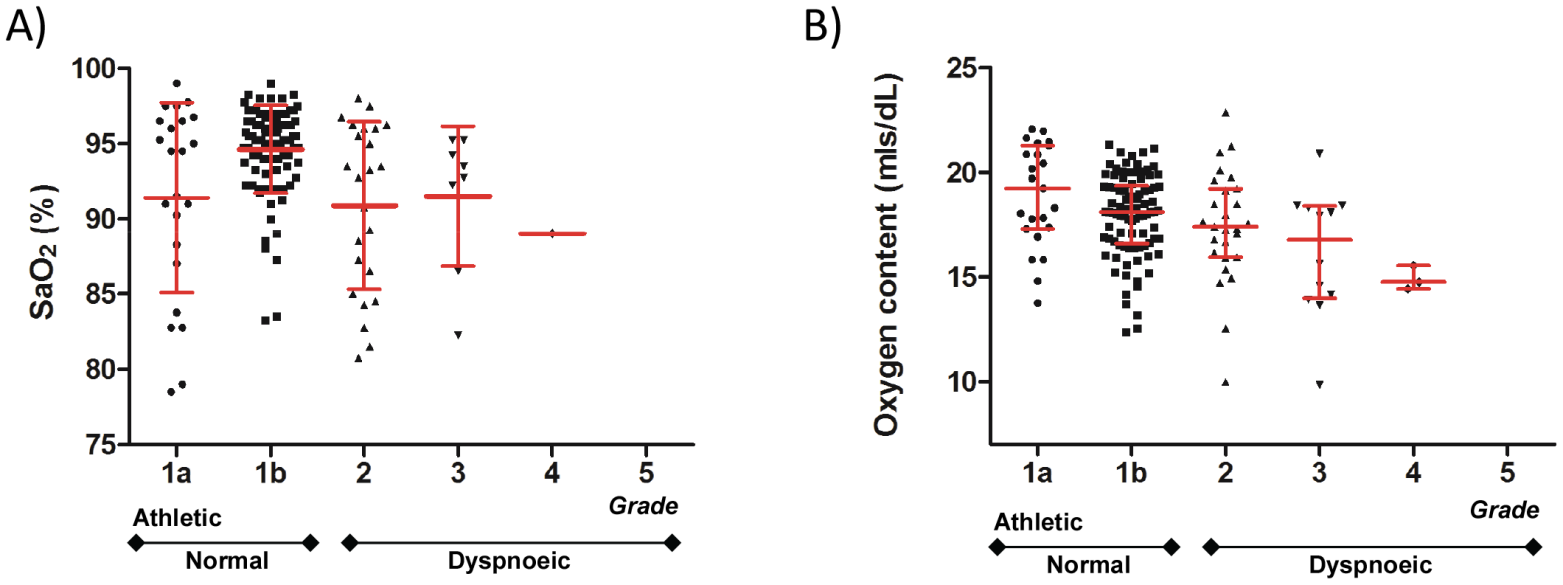
165 PAVM patients graded according to self-reported exercise tolerance. A) SaO_2_ relationships. Error bars represent mean and standard deviation. Similar trends were observed for median and IQR (data not shown). B) Oxygen content, calculated by *SaO_2_ x haemoglobin x 1.34/100*, where SaO_2_ was expressed as a %, and 1.34mls is the amount of oxygen carried per gram of haemoglobin [1]. Error bars represent mean and standard deviation, but a similar trend was observed for median and IQR (data not shown).

Figure 4A demonstrates that there was no clear relationship between SaO_2_ (the most commonly measured index of oxygenation in clinical practice [34] [35],) and graded exercise tolerance: Seven individuals regularly participated in intense sporting activity despite marked resting hypoxaemia (SaO_2_<90%, Figure 1). In contrast there was a very clear inverse correlation between exercise grade and arterial oxygen content, which incorporates both SaO_2_ and haemoglobin. As shown in Figure 4B, exercise tolerance was worse in patients with lower oxygen content (coefficient −0.0012, p<0.0001), with the trend evident across all grades of exercise tolerance.

### Oxygen content and iron relationships in athletes

**Figure 5:**
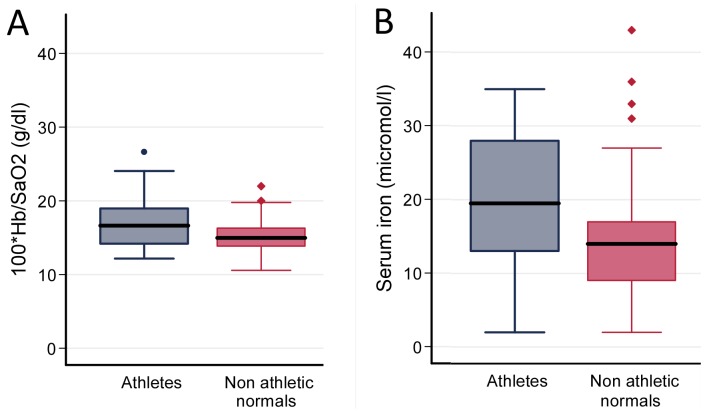
Boxplot comparisons of athletes and other individuals with normal exercise tolerance. Individuals with normal exercise tolerance (Grade 1) were subclassified into Grade 1a (athletes, grey symbols/lines, if participating in intense sporting activity such as rowing, football, distance cycling or gym activities at least three times per week), and Grade 1b (other normals, red symbols/lines, if they described dyspnoea only on strenuous exertion). All assignments were made blinded to physiological parameters [44]. A) Haemoglobin (Hb) adjusted for SaO_2_, presented as (100*haemoglobin)/SaO_2_. Mann Whitney p value  =  0.0059. P values were also calculated by Kruskal Wallis across all exercise grades, when Dunn's post test correction comparing the athletic and non athletic normals gave a p value of<0.05. B) Serum iron. Mann Whitney p value  =  0.010. P values were also calculated by Kruskal Wallis across all exercise grades, when Dunn's post test correction comparing the athletic and non athletic normals gave a p value of<0.05.

Figure 4B suggested that the blood oxygen content may be higher in the pre-defined athletic group than the other Grade 1 normals. Haemoglobin tended to be higher for the degree of hypoxaemia in the athletes, compared to non-athletic normals (Figure 5A). This could not be ascribed to differential use of iron tablets: excluding the 25 iron tablet users (6 athletes, 19 non athletic normals) the higher ratio of [100*haemoglobin]/SaO_2_ in the athletic group persisted (median 16.6 [IQR 14.7, 20.0] vs 15.2 [IQR 12.2, 17.7], p = 0.016). This would be in keeping with the limited evidence suggesting that exercise training can increase total haemoglobin and red cell mass, attributed to stimulated bone marrow erythropoiesis [49].

The current study cohort suggests a potentially relevant change in iron handling: While there was no difference in serum ferritin values between the athletes (median 38 [IQR 19, 77]µg/L) and non-athletic normals (median 38 [IQR 16, 73]µg/L), the athletic group had higher serum iron concentrations (median 19.5 [IQR 13,28]µmol/l compared to 14 [IQR 9,17]µmol/l, p = 0.01 (Figure 5B)). Again, this could not be ascribed to differential use of iron tablets: excluding the 25 iron tablet users, median serum ferritin values in athletes and other normals were very similar at 43.5 [IQR 19, 86]µg/L and 40 [IQR 25, 78]µg/L respectively. However the differences in serum iron concentrations persisted (athletes: median 21 [IQR 13, 28]µmol/l; non athletic normals: 14 [IQR 10,18]µmol/l, p = 0.043).

### Effect of embolisation and correction of hypoxaemia

**Figure 6:**
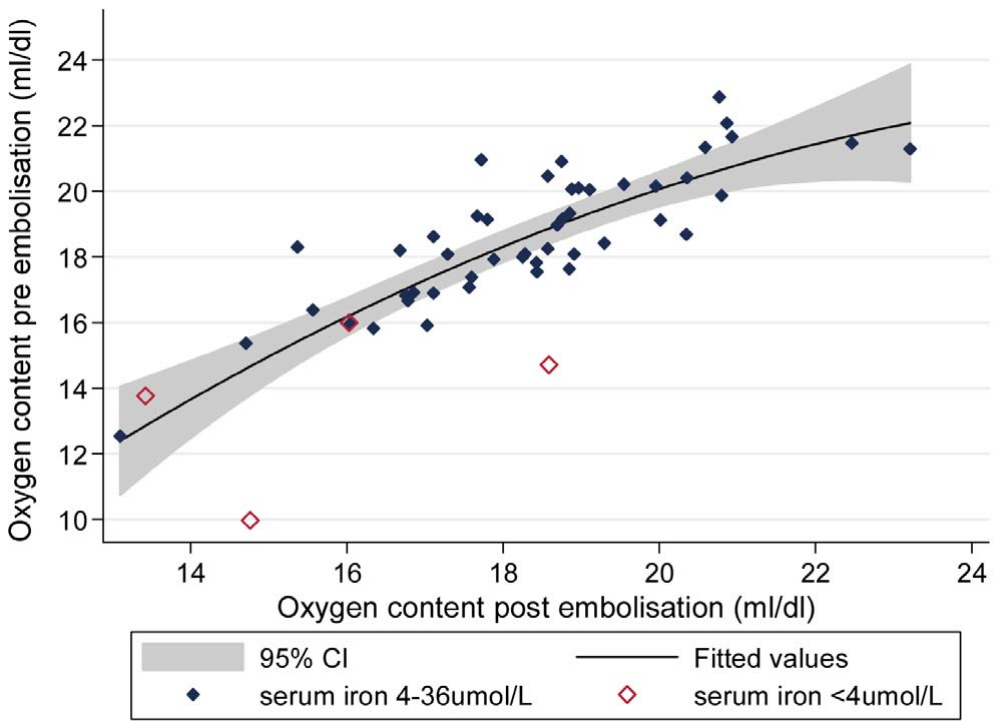
Blood oxygen content pre and post embolisation. Post embolisation data were obtained at clinic follow up at a median of 7 months (range (2–24) months after the final embolisation. Shaded areas represent 95% confidence interval for quadratic regression line for all 52 patients with pre and post embolisation haemoglobin measurements (pseudo r^2^ 0.44, p<0.0001). Open diamonds represent individuals with pre embolisation serum iron <4 µmol/L.

In the 98 patients whose PAVMs were treated by embolisation, SaO_2_ improved immediately (median increase +2.5% (IQR +0.75, +5.0%, Table 2). Improvements were sustained on long term follow up 2–24 (median 7) months later (Table 2). Haemoglobin and haematocrit fell in the follow up period: for haemoglobin, the value at a median of 7 months after final embolisation was 0.60 g/dl lower (IQR −1.3 g/dl, +0.1 g/dl) than at presentation (Table 2). As a result, oxygen content in mls/dl generally returned to pre-embolisation values (Table 2, Figure 6).

**Table 2:** Changes in SaO_2_, haemoglobin, and oxygen content following PAVM embolisation.

	Pre (day -1 or 0)		Immediate post (day 1)		Late follow up (Late post)		Comparison *p* values		
	Median	IQR	Median	IQR	Median	IQR	Pre to day 1	Day 1 to late post	Pre to late post
SaO_2_ (%, N = 71)	94.0	91.8, 95.8	96.3	95.3, 97.0	96.0	95.0, 97.0	<0.001	ns	<0.001
Haemoglobin (g/dl, N = 51)	14.9	13.3, 16.1	.	.	14.3	13.3, 15.2	.	.	0.17
Oxygen content (ml/dl, N = 51)	18.3	16.9, 20.1	19.4	17.4, 20.9	18.4	16.9, 19.3	<0.001	<0.001	ns

Data are provided only where available at all comparative timepoints for stated variable: immediately pre embolisation (previous evening, or same morning); the morning following embolisation (“immediate post (day 1)”); and at late follow up (“late post”), which referred to the first post embolisation follow up clinic, 2–24 (median 7) months post final embolisation. Where several embolisations took place in a series (17 patients required two sessions and 3 required three sessions), data are only reported pre and post final embolisation. The day 1 arterial oxygen content was calculated using the day 1 SaO_2_ and pre-embolisation haemoglobin. P values for three way comparisons were calculated by Friedman, except for two way pre post haemoglobin comparisons which were calculated by Mann Whitney. IQR, interquartile range. ns, non significant (exact figure not provided from Kruskal Walllis.)

In these 98 patients, 86 (87.8%) reported no change in exercise tolerance at their first post-embolisation follow up clinic at a median of 7 months (range 2–24, [IQR 6, 9]) post final embolisation. A common comment was feeling better initially after embolisation, then returning to normal and the individuals not being sure if this was because they had ‘got worse’ again, or become more used to an improved state. We interpreted this as reflecting an interim increase in arterial oxygen content before the fall in haemoglobin documented in Table 2.

Twelve patients (12.2%) did report sustained improvements in exercise tolerance, particularly on stairs, on hills, or during specific sports. Three commented on an improved breathing pattern during swimming (n = 2) or yoga (n = 1). Using logistic regression, there was a trend for improvement in exercise tolerance to be reported more commonly by patients with lower oxygen content at presentation, but no relationship with degree of improvement in oxygen content post embolisation. Improved exercise tolerance was reported more commonly by patients with lower serum albumin, which is a marker of illness/inflammation (attributed to reduced hepatic synthesis, increased albumin catabolism and vascular permeability [50]). An improvement in exercise tolerance was also reported more commonly by patients with higher PAP (Table 3), especially PAP(diastolic) which most closely reflects pulmonary vascular resistance [51]. We concluded that co-existing diseases were associated with less successful compensation to initial hypoxaemia, and improved exercise tolerance post embolisation.

**Table 3:** Demographics, and univariate associations with improvement in exercise capacity post embolisation for the 98 PAVM patients.

	No reported improvement			Improved exercise tolerance			p value*
*Binary variables*	*Total*	*N*	*%*	*Total*	*N*	*%*	
Gender (N and % female)	86	52	60.0	12	7	58.3	0.90
Ever smoked	76	35	46.1	11	12	27.3	0.25
***Continuous variables***	***Total***	***Median***	***IQR***	***Total***	***Median***	***IQR***	
Age (yr)	86	48	38, 60	12	49.8	39.5, 60.5	0.71
Body mass index (BMI)	78	26.4	22.6, 29.0	12	25.4	22.5, 32.5	0.51
SaO_2_ (%)	86	93.6	90.8, 95.5	12	90.1	81.1, 94.5	0.12
Haemoglobin (g/dl)	84	14.9	13.4, 16.1	12	13.7	12.4, 16.3	0.38
Arterial oxygen content (ml/dl)	84	18.3	17.0, 20.1	12	16.4	15.3, 20.0	0.07
Mean corpuscular volume (MCV, fl)	84	88.8	85.5, 91.8	12	88.7	85.3, 94.8	0.92
Serum iron (µmol/l)	74	14.5	9,19	10	15	10, 20	0.79
Transferrin saturation index (%)	74	23	15,31	10	22	6, 35	0.59
Ferritin (µg/L)	45	30	21,67	8	28	11,67	0.76
Albumin (g/L)	72	41.5	39, 44	12	38	36,40.5	0.0086
PAP (systolic)	68	23	20,26	10	26	23,33	0.031
PAP (diastolic)	68	8	7,10	10	9	7,13	0.024
PAP (mean)	68	14	12,16.5	10	16	14,21	0.037
Change in SaO_2_ post embolisation (%)	98	2.38	0.75,5.0	12	4.25	1.375, 7.25	0.54
Change in oxygen content post emb.(ml/dl)	44	−0.2	−1.0, 0.41	9	0.51	−0.08, 1.11	0.14

Patients stratified into those reporting and not reporting improved exercise tolerance. N, number with stated variable; IQR, interquartile range. PAP, pulmonary artery pressure; emb., embolisation. *P values calculated by logistic regression, and shown in bold where <0.05: Key odds ratios (and 95% confidence intervals) were 0.71 (0.56, 0.92) for albumin; 1.12 (1.01, 1.24) for PAP(systolic); 1.31 (1.04, 1.65) for PAP (diastolic); and 1.18 (1.01, 1.40) for PAP (mean). Note inverse associations are indicated by odds ratios <1.

## Discussion

In this study, which examines chronic compensatory changes in an unselected cohort of 165 consecutive patients with PAVMs, we demonstrate that the adaptive polycythaemic/secondary erythrocytotic response not only maintains arterial oxygen content for SaO_2_ ≥78·5%, but also resets to the same arterial oxygen content after correction of hypoxaemia. Impairment of haemoglobin synthesis due to iron deficiency was the key determinant of reduced oxygen content, and reflected the high proportion of PAVM patients who had underlying HHT. Serum iron concentrations rather than ferritin were more predictive of a successful polycythaemic response. Future studies will be required to test if higher serum iron:ferritin ratios are a consequence of athletic training [49] [52], or prerequisite. The preliminary data on improved exercise tolerance following embolisation treatment of PAVMs suggest factors unrelated to iron deficiency are also important in preventing optimal compensations to hypoxaemia.

Strengths of the current study include the large number of patients studied with consistent methodologies, the extremely reduced SaO_2_ present long term, the general absence of confounding hypoxic pulmonary vasoreactivity and inflammation, and the high proportion with significant iron deficiency. The absence of arterial blood gas measurements of PaO_2_ may be considered a weakness, but the reproducibility of the replicate pulse oximetry SaO_2_ measurements [24] render this criticism less important. Not all patients had ferritin measured, but in view of the similar cohorts identified by low serum iron and the ferritin values conventionally used to define iron deficiency [24] [46] [47] [48], we concluded that for the parameters under examination, it was appropriate to use serum iron <11 µmol/L as a marker of an iron insufficient state. Although a single centre study, there was little bias introduced by geography (UK-wide referrals), or type of presentation.

The current data are important for oxygen/polycythaemia guidance for PAVM patients, currently extrapolated from data in other patient groups, particularly patients with COPD where HPV also operates. The guidelines [34] [35] [36] are interpreted as stating that PAVM patients who are hypoxaemic and polycythaemic should have venesection (to reduce plasma viscosity [36]), and oxygen supplementation [34] [35] [36]. For HHT/PAVM patients, it is not high haematocrit/haemoglobin that is associated with venous thromboembolism (VTE) [33] [43], but markers of iron deficiency [33] which would be exacerbated by venesection [36] [53] [54]. Venesection will inevitably result in rebound polycythaemia unless iron deficiency is induced [53] [54]. Supplementary oxygen is generally prescribed to hypoxaemic patients acutely to reduce risks of hypoxic tissue injury [34] [35], and longer term to reduce the risks of developing hypoxic pulmonary hypertension [11] [34] [35] and/or hyperviscosity states [36]. The current study reminds of the normal long-term compensations that help prevent tissue hypoxia. Additionally, multiple studies demonstrate that low SaO_2_/PaO_2_ are tolerated well by PAVM patients at rest [13] [14] [15] [16] [17] [19] [20] [22] [24] [25] [26], on exercise [14] [15] [18] [19], and even in flight when barometric pressure and alveolar oxygen tension fall further [55]. The data in the current manuscript suggest the PAVM patients most likely to benefit from oxygen supplementation would be iron deficient patients with less successful polycythaemic compensatory responses, and this could be tested in future trials.

Taken more broadly, the data reemphasise the importance of combining haemoglobin and SaO_2_ to evaluate the oxygen content of arterial blood. Using a term such as OaHb for arterial oxygen content per unit volume on air, calculated by *SaO_2_ x haemoglobin x 1.34/100*, may serve as an aide memoire. The haemodynamic implications of impaired haemoglobin synthesis are not currently emphasised, most likely attributable to the discrepancy identified by Hébert et al [8], between the copious data in the old physiological literature (summarised in [7]) and paucity in more recent clinical studies. In the current study, average arterial oxygen content was 15% lower in the iron deficient group, implying that unless there were local tissue compensations, cardiac output would need to be approximately 15% higher to deliver the same amount of oxygen to the tissues [1] [7]. The incremental increase would be greater in higher output states, for example on exercise, during sepsis, or for HHT patients with hepatic and/or other visceral AVMs with left-to-right shunting. The crude exercise data presented here suggest limitations in cardiac/circulatory compensatory capacity in the setting of lower blood oxygen content, but this will clearly need to be addressed in targeted prospective studies.

In conclusion, discipline-restricted discussions of haemoglobin or commonly measured oxygen parameters in isolation do not address the more physiologically relevant arterial oxygen content (mls of oxygen per unit blood volume). For hypoxaemic patients, secondary erythrocytosis maintains the oxygen content of blood, provided the bone marrow has sufficient time, function, and iron to respond, and this may be enhanced by athletic training.
